# Thermal Evaluation of Bone Drilling: Assessing Drill Bits and Sequential Drilling

**DOI:** 10.3390/bioengineering11090928

**Published:** 2024-09-16

**Authors:** Sihana Rugova, Marcus Abboud

**Affiliations:** 1 Department of Oral Biology and Pathology, Stony Brook University, Stony Brook, NY 11794, USA; 2 School of Engineering, Stony Brook University, Stony Brook, NY 11794, USA

**Keywords:** bone drilling, infrared thermography, orthopedic surgery, implant dentistry, heat, osteotomy, bone cutting, sequential drilling, pilot drill bit, implant bed preparation, peri-implantitis

## Abstract

Sequential drilling is a common practice in dental implant surgery aimed at minimizing thermal damage to bone. This study evaluates the thermal effects of sequential drilling and assesses modifications to drilling protocols to manage heat generation. We utilized a custom drill press and artificial bone models to test five drill bits under various protocols, including sequential drilling with different loads, spindle speeds, and peck drilling. Infrared thermography recorded temperature changes during the drilling process, with temperatures monitored at various depths around the osteotomy. The results reveal sequential drilling does not eliminate the thermal damage zone it creates (well over 70 °C). It creates harmful heat to surrounding bone that can spread up to 10 mm from the osteotomy. The first drill used in sequential drilling produces the highest temperatures (over 100 °C), and subsequent drill bits cannot remove the thermal trauma incurred; rather, they add to it. Modifying drill bit design and employing proper drilling techniques, such as reducing drilling RPM and load, can reduce thermal trauma by reducing friction. Inadequate management of heat can lead to prolonged recovery, increased patient discomfort, and potential long-term complications such as impaired bone-to-implant integration and chronic conditions like peri-implantitis. Ensuring healthy bone conditions is critical for successful implant outcomes.

## 1. Introduction

In dental implant surgery, sequential drilling has long been a standard procedure due to the understanding of the impact of heat on bone. Research from the 1980s demonstrated that heating a rabbit tibia to above 50 °C for one minute resulted in hemolysis, necrotic lipocytes, and the cessation of blood flow within two days. After seven days, all pre-existing blood vessels had disappeared, and new vessels began to invade the bone. By 30–40 days, 30–40% of the bone had been resorbed and replaced with fat cells. Beyond 40 days, the number of lipocytes increased by 150–200% compared to pre-heating levels. Heating bone to 60 °C or higher resulted in permanent cessation of blood flow and evident bone tissue necrosis, with no signs of repair over follow-up periods of 100 days [1,2,3].

It is well-established that heat generated during bone drilling can cause the types of traumatic tissue responses discussed [1,2,3,4,5,6,7,8,9,10,11,12,13]. Sequential drilling is believed to avoid these consequences by using progressively larger drill bits to expel damaged bone. Typical sequential drilling will start with a 2.0 mm diameter drill bit, often referred to as a pilot drill, followed by multiple drill bits increasing in diameter by 0.2 to 0.7 mm increments. To place an implant with a 4.1 mm diameter, it is common for a surgeon to use a series of four drill bits to achieve the desired osteotomy size.

In this study, we evaluate the thermal effects of incrementally widening an osteotomy through sequential drilling and explore modifications to such drilling protocols to influence heat generation. Initially, we follow the manufacturer’s protocol and subsequently simulate clinical adjustments. Additionally, we investigate the role of the pilot drill in optimizing subsequent drilling sequences.

## 2. Materials and Methods

**Figure 1:**
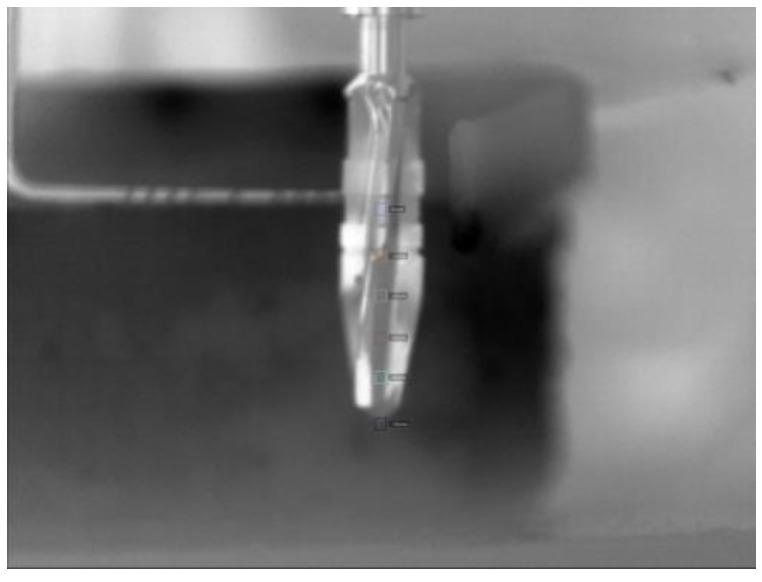
Image of Ø4.1 mm in front of bone–simile taken by infrared camera.

The testing procedure was carried out according to Rugova and Abboud 2024 [14]. A custom-built drill press with a W&H implant motor (W&H Group, Bürmoos, Austria) was used to ensure a standard, unbiased drilling procedure for each osteotomy. Ten-millimeter-deep osteotomies were drilled into artificial bone similes (BS180035-120035-180035, BoneSim, Cassopolis, MI, USA), and a high-precision Computer Numeric Control (CNC) machine was used both to cut the bonesims and to mark each osteotomy site. The bonesim strips were soaked in room temperature saline for 20 min prior to drilling to simulate a more fluid environment. External irrigation at a flow rate of approximately 12 mL/min was used throughout while an infrared camera (FLIR A325sc infrared camera, FLIR Systems Inc., Wilsonville, OR, USA) fixed with a close-up 4× lens acquired temperature data from the surface of the bone 0.5 mm away from the final osteotomy. Figure 1 shows a still image taken with the infrared camera of the largest drill bit used in this study in front of the bone block in monochrome.

**Figure 2:**
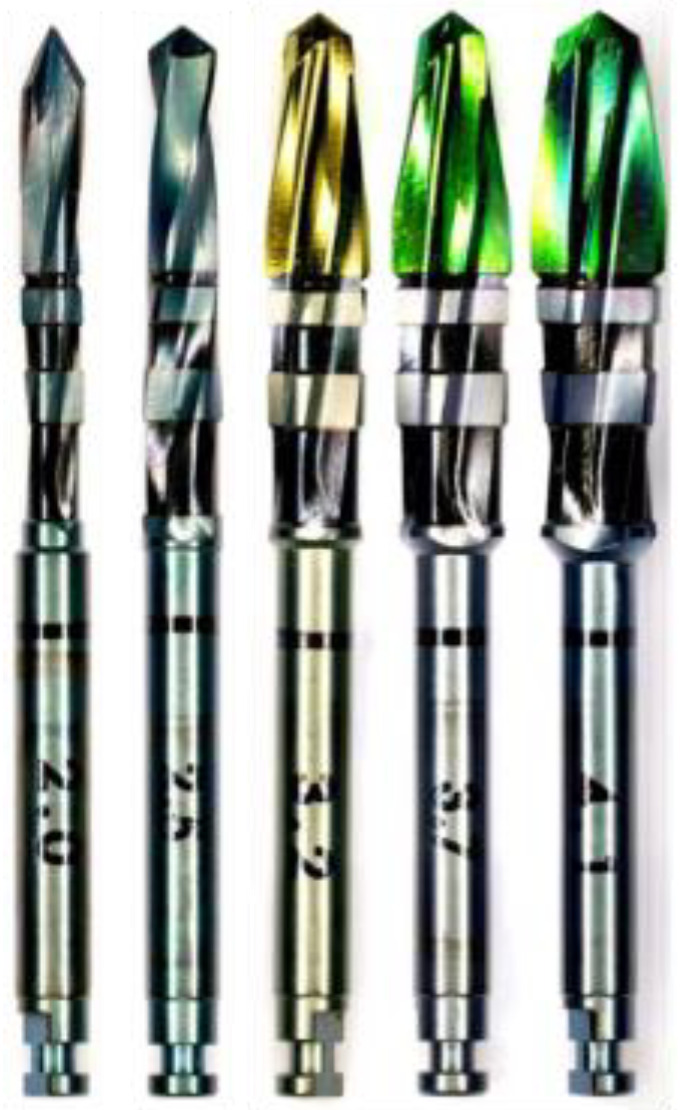
Image of five drill bits used. From left to right: Ø2.0 mm, Ø2.5 mm, Ø3.2 mm, Ø3.7 mm, and Ø4.1 mm.

Five drill bits (Figure 2) donated by a USA-based implant company were tested at two different loads (1.2 kg and 2.3 kg) for three different sequential drilling protocols:

(1) Sequential drilling using the manufacturer’s recommended drill bits and corresponding spindle speeds;
(2) Sequential peck drilling using the manufacturer’s recommended drill bits and corresponding spindle speeds;
(3) Sequential drilling using the manufacturer’s recommended drill bits with a constant spindle speed of 1500 rpm.

In addition, a one-drill protocol was tested using only the 2.0 mm diameter drill bit at two different loads (1.2 kg and 2.3 kg) as the subsequent drills were made to be used sequentially and therefore could not penetrate bone unless excessive force was used to break through the cortical layer of bone. The 2.0 mm drill bit is usually used to initiate the osteotomy, allowing the following drill bits to sequentially widen the osteotomy to the intended diameter.

Peck drilling is completed by drilling to half of the desired depth, removing the drill bit briefly while irrigation is still applied, and then allowing the drill bit to complete the osteotomy to the desired depth of ten millimeters. Table 1 shows the eight different protocols tested and the spindle speeds used for each protocol.

Videos of the osteotomy procedure were recorded in a dynamic rainbow palette (20 colors) using FLIR ResearchIR Max version 1 on Windows 8.1. Temperature readings were recorded continuously before, during, and after the osteotomy procedure to determine the maximum bone temperatures (Celsius) and duration (seconds) of temperature influence. Base temperatures were normalized to 32 °C to match the maximum maxillary temperature recorded in the literature [15].

Statistical Analysis: The data analysis for this study was generated on Microsoft Excel (Microsoft, Washington, DC, USA, Version 16, 2021). The temperatures achieved under each condition were recorded and compared statistically by a Student’s *t*-test. Statistical significance was established as *p* < 0.05; *n* = 10.

Tissue damage is both time- and temperature-dependent. The threshold for irreversible tissue damage in this study was 50 °C for 30 s [3]. If the temperature remains in the range of 50–70 °C for 30 s or longer, the damage produced is irreversible. Temperatures at or above 70 °C indicate immediate osteocyte death regardless of duration [4,16].

Thermal video recordings were analyzed to document the maximum temperature readings at 5 regions of interest, at depths of 0, 2, 4, 6, 8, and 10 mm, 0.5 mm away from the periphery of the osteotomy throughout the duration of the procedure.

## 3. Results

**Figure 3:**
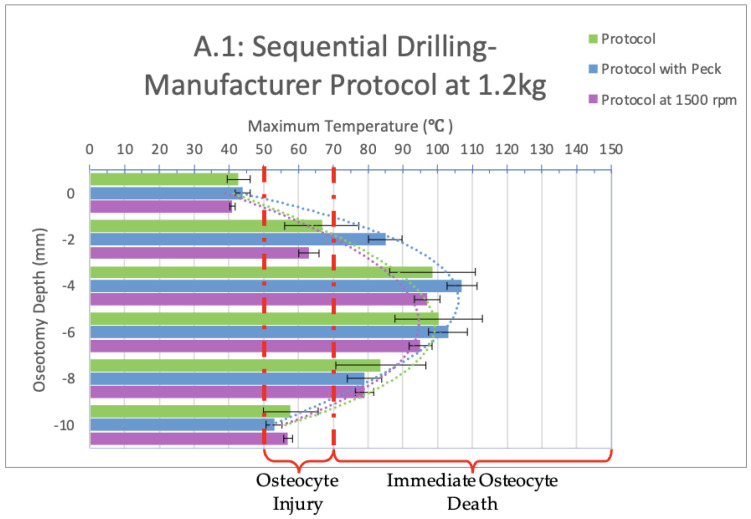
Bar graph showing maximum temperatures reached when drilling into the bone to a depth of 10 mm with a load of 1.2 kg. The temperature range of osteocyte injury and osteocyte death are marked on the graph. The left axis shows the drilling depth (in millimeters), while the top axis shows the maximum temperature (in Celsius) reached at the corresponding depth. Temperatures are normalized to a maximum maxillary temperature (32 °C).

**Figure 4:**
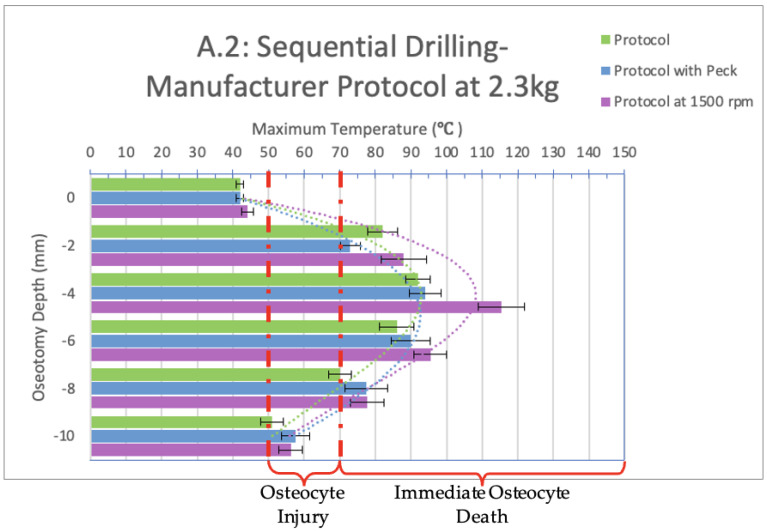
Bar graph showing maximum temperatures reached when drilling into the bone to a depth of 10 mm with a load of 2.3 kg. The temperature range of osteocyte injury and osteocyte death are marked on the graph. The left axis shows the drilling depth (in millimeters), while the top axis shows the maximum temperature (in Celsius) reached at the corresponding depth. Temperatures are normalized to a maximum maxillary temperature (32 °C).

**Figure 5:**
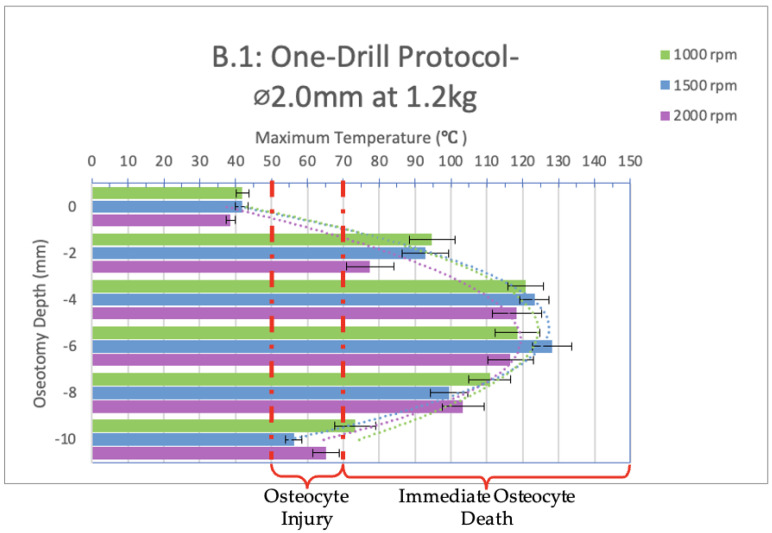
Bar graph showing maximum temperatures reached when drilling into the bone to a depth of 10 mm with a load of 1.2 kg with a 2.0 mm diameter drill bit used at 1000, 1500, and 2000 rpm, respectively. The temperature range of osteocyte injury and osteocyte death are marked on the graph. The left axis shows the drilling depth (in millimeters), while the top axis shows the maximum temperature (in Celsius) reached at the corresponding depth. Temperatures are normalized to a maximum maxillary temperature (32 °C).

**Figure 6:**
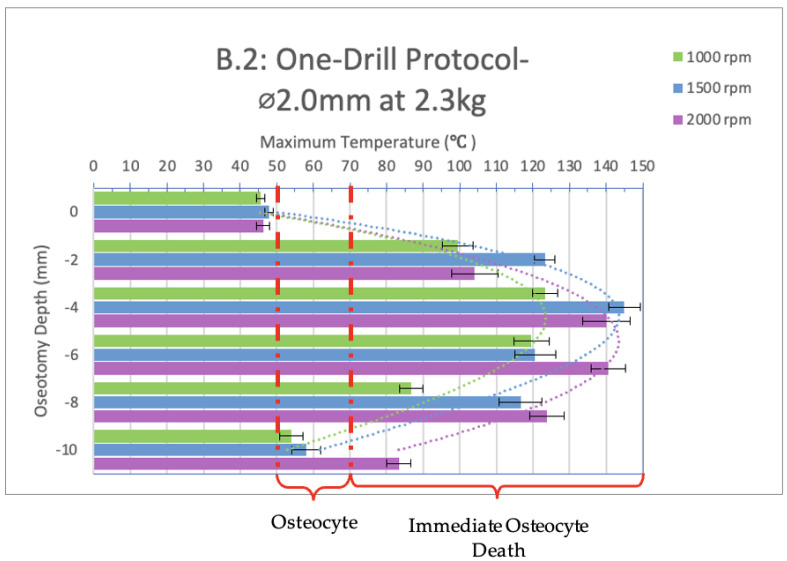
Bar graph showing maximum temperatures reached when drilling into the bone to a depth of 10 mm with a load of 2.3 kg with a 2.0 mm diameter drill bit used at 1000, 1500, and 2000 rpm, respectively. The temperature range of osteocyte injury and osteocyte death are marked on the graph. The left axis shows the drilling depth (in millimeters), while the top axis shows the maximum temperature (in Celsius) reached at the corresponding depth. Temperatures are normalized to a maximum maxillary temperature (32 °C).

Infrared videos taken throughout the osteotomy procedures, as listed in Figure 3, were analyzed, and maximum temperatures at depths of 0, 2, 4, 6, 8, and 10 mm were recorded and organized into the bar graphs shown in Figure 3, Figure 4, Figure 5 and Figure 6 below.

When sequential drilling was performed following the manufacturer’s protocol at 1.2 kg load (Figure 3, group A.1), the only depth that showed a safe temperature range across all three sequential drilling tests (manufacture protocol, manufacturer protocol with peck drilling, manufacturer protocol with constant spindle speed) was at a depth of 0 mm. At a depth of 2 mm, all three groups exceeded the threshold for irreversible damage by temperature (67 °C, 85 °C, 63 °C, respectively) and time (46, 44, 33 s, respectively). At depths of 4, 6, and 8 mm, immediate cell death would be observed in vivo as temperatures well surpassed 70 °C and, at times, 100 °C. At a depth of 10 mm, following the manufacturer protocol, 50 °C was exceeded for 54 s and reached a maximum temperature of 58 °C, indicating irreversible tissue damage. Manufacturer protocol with peck drilling reached a maximum temperature of 53 °C for 17 s, indicating reversible tissue damage, and manufacturer protocol with a constant spindle speed of 1500 rpm exceeded 50 °C for 30 s and reached a maximum temperature of 57 °C, indicating irreversible tissue damage.

A significant difference exists between the peck drilling manufacturer protocol and the other two tests only at a depth of 2 mm.

For all three sequential drilling protocols tested at a 1.2 kg load, these results indicate immediate osteocyte death would be observed in vivo. Clinically, it would be recommended that the implant be left alone to heal for at least three months without interference to allow the recipient bone to recover from the damage caused and undergo the healing necessary for osseointegration to occur. 

When sequential drilling was performed following the manufacturer’s protocol at a 2.3 kg load (Figure 4, group A.2), the only depth that showed a safe temperature range across all three sequential drilling tests (manufacture protocol, manufacturer protocol with peck drilling, manufacturer protocol with constant spindle speed) was at a depth of 0 mm. At depths of 2, 4, 6, and 8 mm, immediate cell death would be observed in vivo as all temperatures reached 70 °C and, at times, 100 °C. At a depth of 10 mm, reversible tissue damage would be observed in vivo for all three sequential drilling tests as the manufacturer protocol exceeded 50 °C for 14 s and reached a maximum temperature of 51 °C; the manufacturer protocol with peck drilling exceeded 50 °C for 20 s and reached a maximum temperature of 58 °C; and the manufacturer protocol with a constant spindle speed of 1500 rpm exceeded 50 °C for 26 s and reached a maximum temperature of 56 °C.

A significant difference is present between the peck drilling and the other two tests at a depth of 2 mm. At 4 mm depth, there is a significant difference between the manufacturer protocol and manufacturer protocol with a constant spindle speed.

For all three sequential drilling protocols tested at a 2.3 kg load, these results indicate immediate osteocyte death would be observed in vivo. Clinically, it would be recommended that the implant be left alone to heal for at least three months without interference to allow the recipient bone to recover from the damage caused and undergo the healing necessary for osseointegration to occur.

When comparing groups A.1 (1.2 kg load) and A.2 (2.3 kg load), a significant difference exists between the manufacturer protocols at depths of 2, 6, and 8 mm; between the manufacturer protocols with a constant spindle speed of 1500 rpm at a depth of 0 and 2 mm; and between the peck drilling manufacturer protocol at a depth of 2, 4 and 6 mm.

Infrared videos taken throughout the osteotomy procedures completed with a one-drill protocol utilizing only the 2.0 mm diameter drill bit were analyzed and are shown below in Figure 5 and Figure 6. The bar graphs show maximum temperatures reached at depths of 0, 2, 4, 6, 8, and 10 mm.

When the 2.0 mm diameter drill bit was used as a stand-alone instrument (one-drill protocol) and not in sequence, with a load of 1.2 kg (Figure 5, group B.1), the only depth that showed a safe temperature range across all spindle speeds tested was 0 mm. At depths of 2, 4, 6, and 8 mm, immediate osteocyte death would be observed in vivo as all temperatures exceeded 70 °C. At depths of 4, 6, and 8 mm, 100 °C was reached or exceeded. At a depth of 10 mm, immediate cell death would be observed in vivo at a spindle speed of 1000 rpm as 50 °C was exceeded for 18 s and reached a maximum temperature of 73 °C. With a spindle speed of 1500 and 2000 rpm, 50 °C was exceeded for 13 and 11 s, while the maximum temperatures reached were 56 °C and 65 °C, respectively. These temperatures indicate reversible tissue damage with delayed wound healing.

A significant difference is present between the 1000 and 1500 rpm spindle speeds at a depth of 10 mm.

When the 2.0 mm diameter drill bit was used as a stand-alone instrument (one-drill protocol) and not in sequence, with a load of 2.3 kg (Figure 6, group B.2), the only depth that showed a safe temperature range across all spindle speeds tested was 0 mm. At depths of 2, 4, 6, and 8 mm, immediate cell death would be observed in vivo as all temperatures well exceeded 70 °C and, at times, 100 °C.

At a depth of 10 mm and spindle speed of 1000 and 1500 rpm, 50 °C was exceeded for 5 and 7 s, while the maximum temperatures reached were 54 °C and 58 °C, respectively. At a spindle speed of 2000 rpm, immediate cell death would be observed in vivo as 70 °C was exceeded.

A significant difference is present between the 1000 rpm spindle speed compared to 1500 and 2000 rpm at a depth of 4 and 8 mm. At a depth of 6 and 10 mm, a significant difference is present between the 2000 rpm test compared to the 1000 and 1500 rpm tests.

When comparing groups B.1 (1.2 kg load) and B.2 (2.3 kg load), a significant difference exists between the 1500 rpm spindle speed at a depth of 4 mm and the 2000 rpm spindle speed at a depth of 0, 2, and 8 mm.

## 4. Discussion

Based on the data collected in this study, we determined that heat damaging to bone cells can spread up to 10 mm in bone from the source of the thermal trauma. This is evident both in Figure 3 and Figure 4, which represent sequential drilling groups A.1 and A.2, as well as in the associated videos. Video S1, an infrared recording from this study, demonstrates the heat produced during sequential drilling for implant bed preparation. The initial use of the 2.0 mm drill bit results in temperatures exceeding 100 °C at a distance of 2.5 mm from the thermal trauma source. Although subsequent drill bits did not reach the same maximum temperatures as the initial drill bit, they still produced heat levels sufficient to cause irreversible tissue damage and immediate cell death. The final drill bit used was Ø4.1 mm, exceeding 80 °C at a distance of 0.5 mm from the source of the thermal trauma. The dissipation of heat becomes more apparent toward the end of the video, where the rainbow color palette vividly illustrates its range and extent. Thus, it is apparent that sequential drilling does not eliminate the thermal damage zone it creates. This, however, is heavily dependent on the design of the drill bit [17,18,19]. The point of a drill bit alone has over 30 anatomical characteristics that can be modified. Adjusting the tip and body design may help prevent thermal trauma.

When comparing the 2.0 mm pilot drill bit to the second drill bit used in the series (Ø2.5 mm), the former has three flutes and a smaller point angle, while the latter is a twist drill containing two flutes and larger point angle, and both have the same general straight body shape that differs by 0.5 mm in diameter. The second drill bit insignificantly increases the temperatures produced, barely showing any difference in the temporal plot in Video S1. The third (Ø3.2 mm), fourth (Ø3.7 mm), and fifth (Ø4.1 mm) drill bits have a similar point angle as the second one; however, they have four flutes, a tapered body shape with the widest portion at 7.5 mm, and an increase of 0.4–0.6 mm in diameter. These three drill bits show a significant increase of about 40 °C on the temporal plot shown in Video S1. Three different drill bit designs show three different temperature profiles when used in series, indicating the importance of design in influencing heat generation during bone drilling.

Video S2, another infrared recording from this study, demonstrates the heat produced during implant bed preparation with the 2.0 mm diameter drill bit in a one-drill protocol. The drill bit not only generated the highest temperatures among all sequential drilling protocols tested, but also showed even higher temperatures when heat was measured 0.5 mm away from the source of the thermal trauma. It is known that as drilling depth increases, the contact time between the drill bit and bone increases, leading to greater temperature-raising friction and, ultimately, higher bone temperatures [20,21,22]. This is evident in the temporal plot shown in both videos. It is clear that if drilling was stopped earlier and the osteotomy was directly and thoroughly irrigated, the recorded temperatures could be reduced. However, our peck drilling results do not show this, as our peck drilling was completed quickly without allowing sufficient time for irrigation fluid to cool the site. Clinically, peck drilling does not allow irrigation fluid to enter the osteotomy, as the drill bit itself is never fully removed from the osteotomy.

To minimize thermal damage during bone drilling, especially in implant dentistry, we recommend that the osteotomy be completed in stages, limiting each drilling depth to a maximum of three times the diameter of the drill bit in one motion. This should be followed by the complete removal of the drill bit from the implant bed and thorough irrigation of the osteotomy for at least 3 s before continuing, in addition to continuous irrigation throughout the procedure. The irrigation fluid should be pre-cooled with sterile saline solution (4 °C, 41 °F), which is a standard temperature for most refrigerators [23,24]. These precautions are essential to reducing, though not entirely avoiding, thermal trauma, as pre-existing design flaws that enhance thermal trauma can never be fully compensated for.

Failure to manage thermal trauma can lead to significant clinical disadvantages and complications. Thermally traumatized bone results in longer recovery times and increased patient discomfort, often necessitating additional pain management interventions. Patients with complex medical histories or those undergoing advanced procedures, such as immediate implant loading, are at an elevated risk of implant failure. Long-term consequences of thermal trauma include a 30–40% replacement of bone with fat tissue, which weakens the bone-to-implant connection by reducing the peri-implant bone density. This weakening is similar to the mechanism by which smoking leads to peri-implantitis [25,26,27,28,29,30]. Given that thermal trauma also compromises the bone–implant interface and bone density, peri-implantitis can be considered an associated significant long-term risk. In addition to the risks present in implant treatments, adjacent teeth and structures may also suffer from conditions such as pulpitis, necrosis, resorption, and sensitivity. These silent threats jeopardize the longevity and success of implant surgeries and patient satisfaction, as any device placed in the bone can only function effectively if surrounded by healthy bone tissue.

## 5. Conclusions

This study underscores the significant thermal impact of sequential drilling in dental implant procedures. In most tests, temperatures exceeded 70 °C, causing irreversible tissue damage and immediate cell death. The pilot drill alone exceeded 100 °F, reaching up to 10 mm away from the osteotomy, farther than the next drill could reach to remove damaged tissue. When drill bit designs do not consider thermal trauma during bone drilling, sequential drilling cannot be used as a surgical technique to eliminate the thermal trauma zone it creates. To improve outcomes, well-engineered drill bits are essential for minimizing heat generation and enhancing precision, working in tandem with refined drilling techniques. Clinical recommendations include limiting drilling depth to two to three times the drill bit diameter per motion and using pre-cooled sterile saline to thoroughly irrigate the osteotomy to reduce thermal trauma. Although surgical techniques can help minimize heat exposure to the bone, they cannot fully compensate for poorly designed drill bits that contribute to excessive heat generation. Effective management of heat during drilling is essential to avoid prolonged recovery and potential complications like peri-implantitis, ensuring better outcomes for dental implant surgeries.

## Figures and Tables

**Table 1:** Table showing drilling groups tested, drill bit diameters used, and spindle speeds used.

Drilling Groups		Drill Bit Diameter	RPM
A.1: Sequential drilling at 1.2 kg A.2: Sequential drilling at 2.3 kg	Sequential drilling— manufacturer recommendation	Ø2.0	2000
		Ø2.5	1500
		Ø3.2	1000
		Ø3.7	1000
		Ø4.1	1000
A.1: Sequential drilling at 1.2 kg A.2: Sequential drilling at 2.3 kg	Sequential drilling— manufacturer recommendation, including peck drilling	Ø2.0	2000
		Ø2.5	1500
		Ø3.2	1000
		Ø3.7	1000
		Ø4.1	1000
A.1: Sequential drilling at 1.2 kg A.2: Sequential drilling at 2.3 kg	Sequential drilling— manufacturer recommendation with constant spindle speed	Ø2.0	1500
		Ø2.5	1500
		Ø3.2	1500
		Ø3.7	1500
		Ø4.1	1500
B.1 One-drill protocol—Ø2.0 mm drill bit at 1.2 kg B.2 One-drill protocol—Ø2.0 mm drill bit at 2.3 kg			1000
			1500
			2000

## Data Availability

Dataset available on request from the authors.

## Supplementary Materials

The following supporting information can be downloaded at: https://zenodo.org/records/13761533. Video S1: Excessive Heat in Sequential Implant Drilling; Video S2: Excessive Heat with 2.0 Implant Drill Bit.
